# Calreticulin Immunohistochemistry in Myeloproliferative Neoplasms - Evolution of a New Cost-Effective Diagnostic Tool: A Retrospective Study with Histological and Molecular Correlation

**DOI:** 10.5146/tjpath.2021.01550

**Published:** 2022-01-21

**Authors:** Sanjeet Roy, Marie Therese Manipadam, Poonkuzhali Balasubramanian

**Affiliations:** Department of Pathology, Christian Medical College, Tamil Nadu, India; Department of Haematology, Christian Medical College, Tamil Nadu, India

**Keywords:** Immunohistochemistry, CAL2, Calreticulin, Myeloproliferative neoplasm, CALR

## Abstract

*
Objective:* Recent WHO 2017 guidelines mandates mutational analysis for the diagnosis of myeloproliferative disorders (MPN). JAK2V617F has been found in only 50-60% of Primary myelofibrosis (PMF) and Essential thrombocythaemia (ET). A recently discovered somatic Calreticulin *(CALR)* mutation has been linked to MPN. This mutation leads to a common 36 amino acid C-terminus that can be detected accurately by immunohistochemistry (IHC). Limited published literature exists on the utility of CAL2IHC as a diagnostic tool. The study aimed to validate the sensitivity and specificity of CAL2IHC for its use as a cost effective and rapid diagnostic tool.

*
Material and Method:* Subjects included 23 patients of MPN (15 PMF, 6 ET, 2 PV (Polycythaemia Vera)), diagnosed between January 2014 to November 2016 with adequate available tissue for histopathological and mutational analysis. Mutational analysis had been performed with Bidirectional Sanger sequencing. CAL2IHC was performed in all cases and the sensitivity and specificity of CAL2 IHC to identify the Calreticulin mutation was evaluated with respect to comparison with the gold standard mutation analysis.

*
Results:* In the 23 MPN patients, CAL2 IHC detected *CALR* mutation with a sensitivity of 95% and a specificity of 100%. Both cases of PV were negative for CAL2IHC. CAL2IHC showed cytoplasmic positivity in ET (2-3+) and PMF (1-3+) with (62-69%) positive megakaryocyte staining. All 6 ET cases and all 14/15 PMF cases were CAL2IHC positive, and these results were concordant with *CALR* mutational analysis.

*
Conclusion:* Anti-CAL2 immunohistochemistry is a specific and a sensitive marker to detect *CALR* mutation. Its’ cost effectiveness and fast results are quite advantageous as compared to molecular analysis.

## INTRODUCTION

Myeloproliferative neoplasms (MPNs) are a heterogeneous group of diseases that have a diverse clinical presentation and a myriad of morphologies. These intriguing disorders have always proven to be a challenge for haematologists and hematopathologists. Recent studies have necessarily aimed at classifying MPNs based on molecular alterations as there is increasing evidence that molecular or chromosomal alterations have a better correlation with clinical presentation, response to therapies, and prognosis rather than conventional morphological classification (1,2). A significant number of gene mutations have been identified in MPNs with *JAK2 *and *MPL* being the major ones by the World Health Organization (WHO) 2007 (3). The *JAK2V617F* mutation has been found to occur in almost 95% of Polycythemia Vera (PV) (4). However, *JAK2V617F* has been found in only 50-60% of Primary myelofibrosis (PMF) and Essential thrombocythaemia (ET) (5).

A significant gap was present that comprised many cases of MPN that do not harbor any of these mutations, but was recently filled by the discovery of Calreticulin (*CALR*) mutation in MPNs (6,7). *CALR* gene mutations are predominantly found in patients with essential thrombocythemia or primary myelofibrosis and are considered to be mutually exclusive with *JAK2* and *MPL*. In spite of the mutational diversity, all the respective mutations have been shown to function via activation of the JAK-STAT pathway (6,7). With regards to diagnostics, the identification of *CALR* mutations is confirmatory for a diagnosis of MPN in *JAK2* and *MPL* wild type patients, presenting with thrombocytosis. Furthermore, its presence has also shown to carry significant prognostic implications in patients with confirmed MPN.

*CALR*-mutated PMF patients were younger than their *JAK2*-mutated counterparts and displayed higher platelet count, lower leukocyte count and longer survival (8). The haemoglobin levels in PMF with *CALR* mutations were less likely to display anaemia or require transfusion (8).

Many studies demonstrated that ET with *CALR* mutations had higher platelet count than that with *JAK2* mutation and a lower incidence of thrombosis (9). Also, *JAK2-*mutated ET has a 29% cumulative risk of progressing to PV whereas polycythemic transformation was not observed in *CALR-*mutated ET.

The* CALR* gene is located in the short arm of chromosome 19 (10). The most commonly reported pathogenic mutations in *CALR* occur in exon 9 and include 52 base pair (bp), 34 bp, and 19 bp deletions and a 5 bp insertion (6,7). All these insertions or deletions finally result in a frame shift mutation. As a result, the new reading frame codes for a characteristic protein C-terminus that is the same across the last 36 amino acids irrespective of the underlying mutation (11). It becomes important to understand that irrespective of the presence of all the different pathogenic mutations, the final end result is a common protein epitope consisting of a similar sequence of amino acids. If mutation-specific immunohistochemistry is directed to the characteristic C-terminus of mutated Calreticulin, it would prove to be a diagnostic screening tool which is cost effective and provide faster results. Vannucchi et al. (11), using a rabbit polyclonal antibody, and Stein et al. (12), using a mouse monoclonal antibody, have previously demonstrated excellent sensitivity and specificity for the diagnosis of all the different types of Calreticulin mutations. The mouse monoclonal anti-mutant Calreticulin antibody (clone CAL2), the same as that was used by Stein et al., has recently become commercially available. Till date there are limited studies (11–16) on the use of Calreticulin immunohistochemistry as a diagnostic tool for myeloproliferative neoplasms.

According to the current WHO Update (17), the gold standard for mutational analysis in MPN is prioritised with initial analysis of *JAK2V617F* mutation and if negative followed by *CALR* mutation. A bone marrow (BM) biopsy is mandatory for diagnosis of MPN. It has been proposed that if immunostaining for CAL2IHC in BM biopsies is validated, it can be conveniently used for identifying patients harbouring *CALR* mutations. Furthermore, considering its feasibility in any routine histopathology laboratory and the lower cost compared with molecular tests, an initial testing for CAL2IHC may supervene an unnecessary molecular *JAK2V617F* analysis thereby reducing the healthcare charges for a patient.

Therefore, we aimed to test the sensitivity and specificity of CAL2IHC in a diagnostic surgical pathology laboratory that would aid in the identification of pathogenic Calreticulin mutations in the routine clinical setting.

## MATERIALS and METHODS

Subjects of myeloproliferative neoplasms fulfilling the inclusion criteria from January 2014 till November 2016 were recovered with the use of key word search of the electronic data bases of the Pathology and Haematology departments of our institution. The inclusion criteria for the study were: a) subjects with biopsy-proven diagnosis of myeloproliferative neoplasm, b) the availability of adequate bone marrow trephine biopsies for immunohistochemistry, c) blood/tissue available and subjected to *CALR/JAK2 V617F/ JAK* Exon 12 mutational analysis.

Following the selection, all formalin-fixed and paraffin-embedded sections were stained in the automated immunostainer in the General Pathology department with CAL2 antibody (clone CAL2, catalogue DIA-CAL100; Dianova, Germany) at a dilution of 1:20, and according to the protocol T40 in the Ventana Benchmark automatic immunostainer, keeping in accordance with the steps mentioned as per the protocol. The investigators were blinded to the mutation status when examining the slides. To confirm the pathological evaluation, all biopsies were reviewed by 2 pathologists (SR and MTM).

Positive immunohistochemical staining of CAL2IHC was defined by the presence of any intensity (grade 1-3+) of cytoplasmic staining of megakaryocytes. If a tissue section contained more than 50 megakaryocytes, then a total of 50 megakaryocytes were counted. From this count, the number of megakaryocytes staining positively for CAL2IHC (1+ to 3+ staining intensity) were counted separately. Following this, the total percentage of CAL2 positive megakaryocytes was calculated from it (CAL2 positive megakaryocytes / 50) X100. If a section contained less than 50 megakaryocytes, then the total numbers of megakaryocytes present in a section were counted, and from among them the percentage of CAL2 positive megakaryocytes (CAL2 positive megakaryocytes / total megakaryocytes counted) X100 was calculated accordingly. The intensity of cytoplasmic staining for CAL2IHC in megakaryocytes was graded from 1+ weak positivity to 3+ strong positivity.

*CALR* gene deletions and insertions were tested by capillary electrophoresis (gene scan analysis) and the positive cases were confirmed by bidirectional Sanger Sequencing to identify the type of mutation using published protocols (6,7). The sensitivity and specificity of CAL2IHC positivity in patients with MPN were calculated and the results compared with the gold standard molecular analysis for validation as a rapid diagnostic tool.

Clinical information of the patients was collected and histological evaluation and review of CAL2 positive bone marrow trephine biopsies were also done for confirmation of the diagnosis. The parameters evaluated were as follows: a) Cellularity, b) Erythroid hypoplasia, c) Megakaryocyte hyperplasia, d) Presence of giant hyperlobulated cells, e) Presence of small to intermediate size megakaryocytes, f) Nuclear abnormality of megakaryocytes, g) Clustering and paratrabecular location of megakaryocytes, h) Reticulin fibrosis (WHO 2008 grading), i) Vascular proliferation, and j) Osteosclerosis.

All procedures performed in the current study were approved by the Institutional review board (IRB Min no.10587, date 29/3/17) in accordance with the 1964 Helsinki Declaration and its later amendments. Formal written informed consent was not required with a waiver by the institutional review board committee.

## RESULTS

A total of 23 subjects with adequate bone marrow trephine biopsies and peripheral blood sample available for mutational analysis were included in the study. There were 19 males and 4 female patients, aged (30-65) years. The cohort included 15 patients with diagnosis of primary myelofibrosis, 6 with essential thrombocythaemia, and 2 with Polycythemia Vera. Detailed clinicopathological features were described in Table 1.

**Table 1:** Clinicopathological characteristics of myeloproliferative neoplasm cases with JAK2 and CALR Mutational status with CAL2IHC.

	**Essential Thrombocythaemia**	**Primary Myelofibrosis**
**Age (years)**		
Median	36	57
Range	30-58	38-65
Interquartile range	30-48.2	38-49.5
**Male:Female ratio**	6:0	5:1
**Haemoglobin (gm/dl)**		
Median	14.15	9
Range	11.5-15	4.7-11.4
Interquartile range	11.5-14.7	4.7-7.35
**Total leucocyte count (per cubic mm)**		
Median	9,800	11,600
Range	5,066-14,100	1800-48,200
Interquartile range	5,066-13,275	1800-7,500
**Platelet count (per cubic mm)**		
Median	8,21,000	2,46,000
Range	8,000-14,40,000	8,000-8,95,000
Interquartile range	8,000-11,99,500	8,000-1,22,500
**Lactate dehydrogenase (LDH**)		
Median	852.3	1525.1
Range	596-9175	871-3,896
Interquartile range	596-2979.2	746.2-1121
**Histopathology**		
Cellularity (increased/decreased)	6/6 (100%) Increased	9/15 (60%) Increased
Erythroid hypoplasia	0	10/15 (66%)
Granulocytic hyperplasia	0	10/15 (66.7%)
Megakaryocyte hyperplasia	6/6 (100%)	11/15 (73%)
Giant hyperlobulated	6/6 (100%)	0
Small to intermediate sized megakaryocytes	1/6 (17%)	12/15 (80%)
Megakaryocyte nuclear dysplastic abnormality	0	100%
Clustering/paratrabecular location of megakaryocytes	0	100%
Grade 3 reticulin fibrosis	0	100%
Vascular proliferation	0	13/15 (86%)
Osteosclerosis	0	14/15 (93.3%)
**CAL2 Immunohistochemistry**	6/6(100%)	14/15 (93%)
Megakaryocyte staining (%, Range)	69% (20-100%)	62% (25-90%)
Average intensity (1-3+)	2-3+	1-3+
**Molecular analysis**		
*Calreticulin*	6/6 (100%)	15/15 (100%)
*JAK2 Exon 12*	0	0
*JAK2 V617F*	0	0

### Analysis of CAL2 Immunohistochemistry (IHC)

The histopathological diagnosis, CAL2 IHC results, and correlation with the mutational analysis were described in Table 2.

**Table 2:** Pathological analysis of CAL2IHC and comparison with mutational analysis.

**Myeloproliferative Neoplasms**	**Positive Calreticulin Immunohistochemistry**	**Mutational Analysis**		
		***Calreticulin***	***JAK2 Exon 12***	***JAK2V617F***
ETa	6/6 (100%)	6	0	0
PMFb	14/15 (93%)	15	0	0
PVc	0/2 (0%)	0	1	1

**a:** Essential thrombocythaemia, **b:** Primary Myelofibrosis, **c:** Polycythaemia Vera.

All the patients in our cohort had undergone mutational analysis.

**Figure 1:**
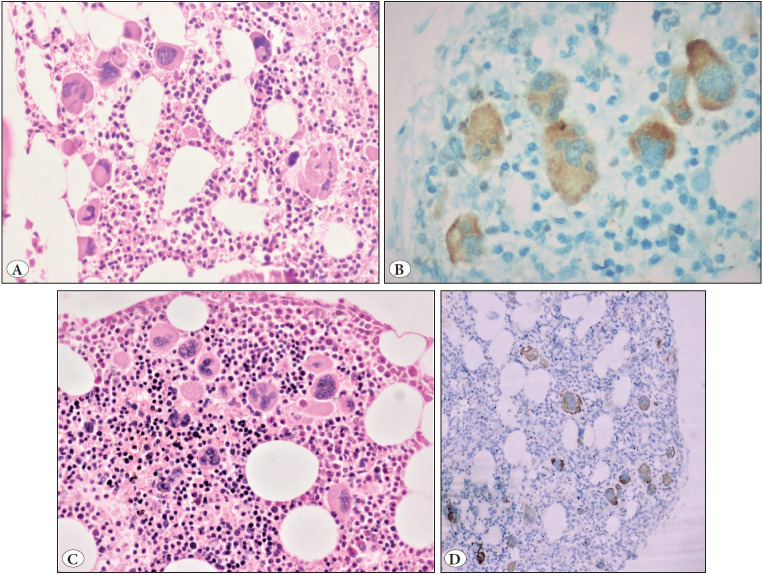
**A-D)** Bone marrow trephine biopsy in essential thrombocythaemia; Patient 1: **A) **Increased number of giant hyperlobulated megakaryocytes, Haematoxylin and Eosin, 200x magnification. **B)** CAL2IHC (2-3+ intensity) diffuse positive cytoplasmic staining of megakaryocytes in essential thrombocythaemia, 200x magnification; Patient 2: **C)** Aggregate of hyperlobulated megakaryocytes, Haematoxylin and Eosin, 200x magnification, **D)** Diffuse cytoplasmic staining of megakaryocytes for CAL2IHC(2-3+ intensity), 100x magnification.

All 6 cases of ET (Figure 1A-D) in our study showed strong cytoplasmic (2-3+) staining of CAL2IHC, displaying 69% (20-100%) positive megakaryocyte staining and showing complete concordance with molecular analysis. Only 1 case showed 1+ positivity, whereas 3 cases showed 2+ positivity, and 2 cases showed 3+ positivity.

**Figure 2:**
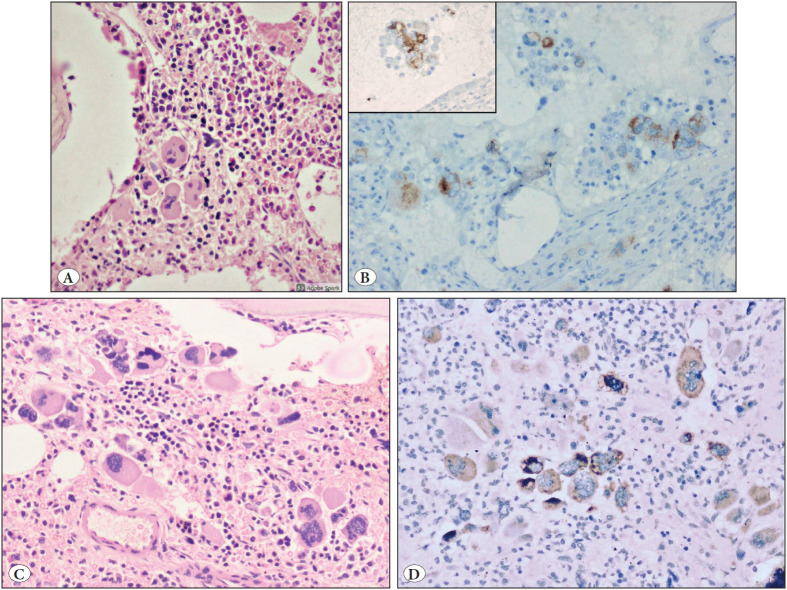
**A-D)** Bone marrow trephine biopsy in primary myelofibrosis **A-D) **Patient 1: **A)** Clustering of dysplastic megakaryocytes, Haematoxylin and Eosin, 100x magnification, **B)** Cytoplasmic staining of megakaryocytes (1-2+ intensity) by CAL2IHC. Inset shows cytoplasmic staining of a dysplastic megakaryocyte, 200x magnification; Patient 2: **C)** Increased number of dysplastic megakaryocytes, demonstrating clustering, abnormal clumped nuclear chromatin, anisocytosis and high N:C ratio, Haematoxylin and Eosin, 200x magnification. **D)** CAL2IHC (1-2+ intensity) positive cytoplasmic staining in megakaryocytes in primary myelofibrosis, occasional megakaryocytes are negative for CAL2IHC, 200x magnification.

14/15 cases of PMF (Figure 2A-D) showed (1-3+) staining of CAL2 IHC (Figure 2B), with 62% (25-90%) of megakaryocytes showing positive staining. 5 cases showed 1+ positivity, 6 cases showed 2+ positivity, and 3 cases showed 3+ positivity. One case was negative for CAL2 IHC but was positive for the Calreticulin mutation. This continued to be negative even on repeated immunohistochemistry preparations.

**Figure 3:**
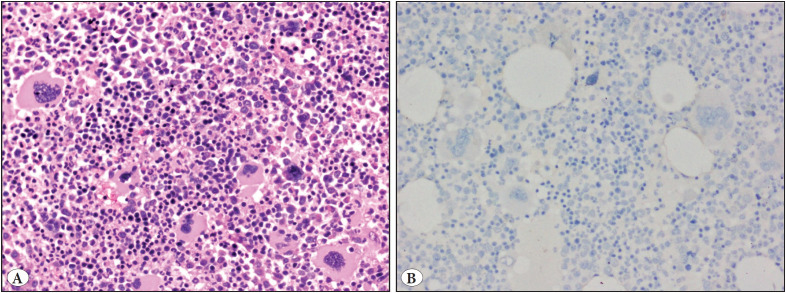
**A,B)** Bone marrow trephine biopsy in Polycythemia Vera: **A)** Panmyelosis with erythroid and megakaryocyte lineage predominance along with absence of stromal fibrosis, Haematoxylin and Eosin, 200x magnification, **B)** The megakaryocytes are negative for CAL2IHC, 200x magnification.

Two cases of Polycythemia Vera (Figure 3A,B) were negative for CAL2IHC, and were also concordant with negative Calreticulin mutation. One of these two patients was positive for the *JAK2* Exon 12 mutation, and the other one was positive for the *JAK2V617F* mutation.

CAL2IHC had a sensitivity of 95.2% and specificity of 100% for effective diagnosis of Calreticulin positive MPN.

### Histopathological Analysis

Histological findings in the bone marrow trephine biopsies of 21 CAL2 IHC positive cases (15 PMF/6 ET) were reviewed. Upon evaluation of 15 cases with PMF, 9/15 (60%) cases showed increased cellularity, 10/15 (66.7%) had granulocytic hyperplasia, 11/15 (73%) had megakaryocyte hyperplasia, with predominantly small megakaryocytes in 12/15 (80%) patients. All cases showed clustering, paratrabecular location with Grade 3 reticulin fibrosis. 13/15 (86%) showed vascular proliferation and 14/15 (93.3%) cases showed osteosclerosis.

All the 6 cases with ET showed increased bone marrow cellularity and megakaryocyte hyperplasia with giant hyperlobulated megakaryocytes. 4/6 (66.7%) cases had 1+ reticulin fibrosis and the remaining 2 cases showed Grade 1 to focal grade 2+ reticulin fibrosis. None of the cases demonstrated any evidence of vascular proliferation or osteosclerosis.

## DISCUSSION

The detection of the Calreticulin mutation has been time proven to carry prognostic value. The importance of its detection also lies in the confirmation of a diagnosis of MPN. On many occasions both clinically and histologically, it becomes extremely difficult even to separate a benign reactive phenomenon from MPN.

Recently, a number of reports have come up highlighting the concurrent presence of multiple MPN related mutations (*JAK2V617F*, *MPL* Exon 10 or *CALR*) with concurrent *BCR-ABL* positive Chronic Myeloid Leukemia (18,19). These reports show that a complex admixture of different clonal population of cells with varying mutations can possibly exist together in the same patient.

The postulations for such a phenomenon are as follows. Firstly, a particular clone by gradual evolution progressing to show distinct different mutations (20). Secondly, the presence of two independent clones in different proportions right from the beginning of disease manifestation. Targeted therapy driven towards a particular clonal population (mostly Ph +v CML) may suppress the former population and facilitate the emergence of the other relatively masked clonal (*CALR* mutated) population (19,20). There are few reports showing that further research with detailed molecular studies is warranted to uncover such hidden anomalies in patients with atypical presentations of MPN.

Considering such complex situations, the mutational analysis often serves as an important confirmatory marker in the diagnosis of a neoplasm (13). Currently molecular testing is the gold standard for identification of *CALR* mutation (17).

Recently, the use of CAL2IHC has been reported to serve as an effective marker for detection of Calreticulin mutations.

Currently, there is only limited reported data on the sensitivity/specificity of this novel antibody and its effectiveness as a diagnostic tool (11–16) (Table 3). Our study aimed to validate the utility of CAL2IHC in routine diagnostics, which in the long run could possibly serve as a surrogate diagnostic tool for molecular studies.

**Table 3:** Comparative analysis of sensitivity and specificity of CAL2IHC.

**Authors**	**Sample size**	**Sensitivity**	**Specificity**
Vannucchi et al. (11)	20	100%	100%
Stein et al. (12)	173	100%	100%
Andrici et al. (13)	29	82-91%	100%
Nomani et al. (14)	38	100%	100%
Mózes et al. (15)	117	100%	100%
Gupta et al. (16)	31	89.4%	100%
Present study	23	95%	100%

Among the 6 reported studies, the first study was done by Vannucchi et al. (11) where a novel polyclonal antibody was developed against all different *CALR* mutations. The antibody was found to be extremely effective with 100% sensitivity and specificity. There was predominant cytoplasmic staining of megakaryocytes and weaker faint staining of erythroids. This was postulated to be due to over expression of *CALR* mutant protein in megakaryocytes. Our study did not show any positive staining of the erythroids or myeloids, and demonstrated a crisp cytoplasmic positive staining of megakaryocytes, therefore concurring with the proposed postulation of over expression of mutant Calreticulin in megakaryocytes.

Subsequently the largest study was performed by Stein et al. (12) where a monoclonal antibody was tried on 173 subjects. The subjects included 155 patients with MPN and the results of immunohistochemistry were compared to the gold standard Sanger sequencing for *CALR* mutation. A high sensitivity and specificity of 100% was quoted in the study. A study on 38 subjects by Nomani et al. (14) also showed a sensitivity and specificity of 100%. A recent study by Andrici et al. (13) showed a mildly lower sensitivity of 91%. Our study similarly showed a sensitivity of 95.2%, and specificity of 100%.

One patient in our study with a diagnosis of PMF was consistently negative for CAL2IHC even after repeated immunohistochemical staining. This observation was also noted by Andrici et al. (13) where a case of PMF was persistently negative for CAL2IHC. Although a possibility of true negative could not be predicted accurately, it was postulated that in end stage cases of PMF, the extensive fibrosis could mask the neoplastic clone population (CAL2 IHC positive staining) of megakaryocytes. In such a situation, only the non-neoplastic population of (CAL2 IHC negative) megakaryocytes may remain more relatively exposed and visible. Hence, based on this observation, the biopsy could be falsely interpreted to be negative for Calreticulin mutation.

This observation could certainly apply for our case as there was extensive fibrosis with paucity of megakaryocytes in the trephine biopsy. The patient was eventually lost to follow up and a repeat biopsy could not be performed. The other possibility was of a false positive result on the mutational analysis. This was difficult for us to evaluate further as there was insufficient tissue for a repeat molecular analysis.

If we consider this to be a true negative, it becomes imperative to realise that a negative CAL2IHC may not always predict negativity for *CALR* mutation. This fact is justified well by Andrici et al. (13) and also by our study.

The next part of our study focused on the morphometric assessment of CAL2IHC positivity on megakaryocytes. Our study showed 69% positive megakaryocyte staining both in ET and PMF cases. Both the cases of PV were negative for CAL2IHC. Mózes et al. (15) in their study had also performed a manual and automated morphometric analysis and correlated it with the *CALR* mutation load. 45.7% (±2.6) of the megakaryocytes had demonstrated a moderate to strong *CALR* expression manually, and 68.5% (±1.28) of the megakaryocytes by automated analysis. It was also shown that the percentage of megakaryocytes with moderate to strong staining had a positive correlation with higher *CALR* mutation loads. Our study demonstrates a higher proportion of megakaryocytes (83% (2-3+ intensity) in cases of ET, and 60% (2-3+ intensity) in cases of PMF) with moderate to strong CAL2IHC staining. We could not do a detailed mutational load analysis due to financial constraints. It remains to be discovered on a larger scale study whether or not the proportion/ staining intensity of megakaryocyte staining could indeed indicate a higher mutation load and therefore be prognostically significant.

Molecular analysis from peripheral blood is non-invasive and indeed provides more accurate results than IHC on bone marrow trephine biopsies. However, the cost of molecular detection via bidirectional Sanger sequencing is higher than the cost of a single immunohistochemical marker and most importantly requires a high level of technical expertise. Therefore, the need of the hour is a cost effective, sensitive and specific diagnostic test that may aid in substituting the need for molecular diagnostics. This situation becomes extremely important in centers where a set up for extensive molecular testing is not available for routine diagnostics.

A novel approach to the step wise diagnosis of MPN has been recently proposed by Vanucchi et al. (11) where instead of the step wise mutational analysis starting with *JAK2* mutation, CAL2IHC can be done. If the CAL2IHC is positive, it essentially excludes the positivity of *JAK2*, *MPL* and other mutations (7). It also becomes important to understand from a different perspective that the current WHO update (17) mandates the histopathological analysis of bone marrow trephine biopsy, as a major criterion for diagnosis of MPN. So needless to say, it becomes feasible, time saving and cost effective for both patient and clinician to perform immunohistochemistry with faster accurate results. Hence in small health care centers, the role for molecular mutational analysis can be considered as a secondary supporting diagnostic test for discrepant cases instead of a mandatory primary test. Whether it stands the test of time to completely substitute the present gold standard of molecular testing is yet to be seen.

In summary, we conclude that CAL2IHC is rapid, cost effective and highly specific for detecting *CALR* mutation, and is an effective diagnostic tool for diagnosis of MPN.

Our study had limitations that could not be eliminated due to financial constraints. Firstly, our sample size was limited to 23 patients with a selection bias (primarily based on cases which had a sample available for molecular analysis). A larger sample size with varying population could have highlighted the specificity more accurately.

Secondly, CAL2IHC was not performed on normal/non MPN subjects. Due to limited resources and infrequent molecular testing of patients, our study was primarily focused on *JAK2* negative and *CALR* positive MPN. Thirdly, our cohort of PMF did not include cases of prefibrotic stage of- PMF that histologically can very often be a close mimicker of ET. Finally, a detailed gene sequence analysis could not be performed to locate the exact base pair deletion in *CALR* mutation. This could have helped us to understand the specificity of CAL2IHC better as it is reported to be positive in all the different types of *CALR* mutations.

## Conflict of Interest

The authors declare no conflicts of interest.
